# Genetic covariance analysis identifies TMEM106B, ACE and ERC2 as genetic loci shared between Alzheimer's disease and primary psychiatric disorders

**DOI:** 10.1002/alz70855_106714

**Published:** 2025-12-24

**Authors:** Ajneesh Kumar, Nicholas R. Ray, Jiji T. Kurup, Pamela Del Rosario, Masood Manoochehri, Colin Stein, Alyssa N. De Vito, Brenna Cholerton, Robert Sweet, Michael L Cuccaro, Gary W Beecham, Edward D. Huey, Christiane Reitz

**Affiliations:** ^1^ Columbia University Irving Medical Center, New York, NY, USA; ^2^ Department of Psychiatry and Human Behavior, Alpert Medical School of Brown University, Providence, RI, USA; ^3^ Brown University Medical School, Providence, RI, USA; ^4^ Department of Pathology, Stanford University, Stanford, CA, USA; ^5^ Department of Psychiatry and Neurology, University of Pittsburgh, Pittsburgh, PA, USA; ^6^ Dr. John T. Macdonald Foundation Department of Human Genetics, University of Miami Miller School of Medicine, Miami, FL, USA; ^7^ John P. Hussman Institute for Human Genomics, University of Miami Miller School of Medicine, Miami, FL, USA; ^8^ Department of Biostatistics and Data Science, Wake Forest School University of Medicine, Winston‐Salem, NC, USA

## Abstract

**Background:**

Neuropsychiatric Symptoms (NPS) (e.g., aggression, psychosis, anxiety, apathy, depression, agitation, sleep disturbances, repetitive behaviors) occur in 85% of Alzheimer's Disease (AD) patients, and are associated with accelerated decline and greatly increased suffering of patients and their caregivers. Our understanding of the etiology of NPS in AD is inadequate, with treatments for NPS often being ineffective and associated with serious adverse effects. Pharmacological treatments have generally been borrowed from their indications for psychiatric illness, despite limited understanding to what extent the molecular and genetic architectures underlying AD‐associated NPS overlap with that of primary psychiatric disorders.

**Method:**

To better characterize the genetic overlap between AD and major psychiatric disorders and identify potentially shared biological processes, we conducted local genetic covariance analyses between AD and each NPS trait using MiXeR and LAVA, capitalizing on the largest most recent GWAS summary statistics for these traits (AD: *n* = 487,511; bipolar disorder: *n* = 413,466; depression: *n* = 1,154,267; schizophrenia: *n* = 130,644, anxiety: *n* = 1,096,218). Identified overlapping genomic loci were followed up with fine mapping, in silico functional analyses, and sequence data analyses from the ADSP.

**Result:**

Local genetic covariance analyses identified four loci shared between AD and depression, five loci shared between AD and schizophrenia, three loci shared between AD and bipolar disorder, and four loci shared between AD and anxiety. Functional fine mapping and sequence data analyses at these loci pinpoint a missense variant in *TMEM106B* shared with both depression and anxiety in the same haplotype as previously reported in FTLD and hippocampal sclerosis, a regulatory region variant in *ACE* as a causative variant shared with schizophrenia, and two distinct haplotypes in *ERC2* carrying NMD transcript variants associated with AD and anxiety, respectively. *TMEM106B* is involved in lysosomal trafficking, *ACE* cleaves several neuropeptides including Aβ‐derived peptide substrates, *ERC2* is involved in neurotransmitter release.

**Conclusion:**

These findings support the notion of pleiotropic overlap between AD and depression, schizophrenia, anxiety respectively, and pinpoint *TMEM106B, ACE*, and *ERC2* as shared genetic drivers. Further functional characterization of these loci, identified sequence variants, and molecular pathways will be critical to characterize the specific underlying biological processes.

